# A Retrospective Study on the Eye-Related Quality of Life, Functional Vision, and Their Determinants Among Children Following Congenital and Developmental Cataracts Surgery and Its Impact on Their Families Using the PedEyeQ

**DOI:** 10.3389/fpubh.2022.788384

**Published:** 2022-03-17

**Authors:** Siyi Gu, Yiwen Hu, Yinying Zhao, Lulu Chen, Weijie Sun, Pingjun Chang, Dandan Wang, Yune Zhao

**Affiliations:** ^1^Eye Hospital and School of Ophthalmology and Optometry, Wenzhou Medical University, Wenzhou, China; ^2^National Clinical Research Center for Ocular Diseases, Wenzhou, China; ^3^Eye Hospital of Wenzhou Medical University Hangzhou Branch, Hangzhou, China

**Keywords:** congenital and developmental cataracts, quality of life, functional vision, PedEyeQ, determinants

## Abstract

**Objectives:**

To evaluate the eye-related quality of life (ER-QOL), functional vision, and their determinants in children following congenital and developmental cataract surgery, as the impact on their families, using the Pediatric Eye Questionnaire (PedEyeQ).

**Materials and Methods:**

This was a retrospective cross-sectional study involving 107 children (aged 0–11 years) with congenital and developmental cataracts who had undergone surgery, as well as 59 visually healthy controls (aged 0–11 years). One parent for each child completed either the Proxy 0–4 PedEyeQ, the Proxy 5–11 PedEyeQ, or the Parent PedEyeQ, depending on their child's age. Mann-Whitney U and Kruskal-Wallis tests were then conducted to compare the differences between groups and to analyze their determinants.

**Results:**

PedEyeQ scores were found to be lower in postoperative children with congenital and developmental cataracts compared with the control group across all study domains. The PedEyeQ Proxy 0 to 4 years' greatest mean difference was 27 points worse in the Functional Vision domain (95% CI −34 to −19; *p* < 0.001). We also found that the occurrence of nystagmus (*p* < 0.005) and strabismus (*p* < 0.005) were the major factors affecting participants' functional vision. The PedEyeQ Proxy 5 to 11 years' greatest mean difference was 23 points worse in this same domain (95% CI −30 to −15; *p* < 0.001), with nystagmus (*p* < 0.05) being the main determinant herein. Parent PedEyeQ 0 to 4 years' greatest difference was 46 points worse on the Worry about their Child's Eye Condition domain (95% CI −57 to −36; *p* < 0.001). Similarly, parents of children with ophthalmologic abnormalities, including nystagmus (*p* < 0.001) and strabismus (*p* < 0.05), were significantly more worried about their children's eye condition. Parent PedEyeQ 5 to 11 years' greatest difference was also found to be 30 points worse on the Worry about their Child's Eye Condition domain (95% CI −43 to −17; *p* < 0.005).

**Conclusions:**

Children who have undergone congenital and developmental cataract surgery experience a lower quality of life and reduced functional vision. Their families are also significantly and adversely affected herein. Thus, more attention is needed on these groups, with more focused measures being administered to both children and their families.

## Introduction

The incidence of congenital and developmental cataracts is 0.4–8.5 per 10,000 children, and may actually be higher according to some research (1). Furthermore, it is the leading cause of childhood blindness, with the percentage of pediatric cataracts causing childhood blindness being 12–39% (2). Additionally, there is a long-term risk of postoperative complications for this condition. Furthermore, long-term treatment, involving follow-ups and rehabilitation post-surgery, is also necessary. Thus, pediatric cataracts can cause significant psychological, financial, and social problems for both the affected children and their families.

Although congenital and developmental cataracts are associated with reduced eye-related quality of life (ER-QOL) (3), the daily impact of the surgery for these conditions on children and their families is poorly understood. Furthermore, the effects of congenital and developmental cataracts on patients' functional vision (4) have not been rigorously studied because of a lack of validated eye-related measurements. Therefore, we used the Pediatric Eye Questionnaire (PedEyeQ), which is a Rasch-scored, child-and-parent-derived, and an age-specific measure of ER-QOL, as well as including a functional vision questionnaire. It was designed in 2019 for use among children with eye-related conditions and their parents by a pediatric eye disease investigator group (5, 6). In our secondary analyses, we found some additional influencing factors that have not been previously reported in either patients' basic or their surgical information.

## Methods

Institutional Review Board approval for this retrospective cross-sectional study was obtained. All data collection and procedures adhered to the tenets of the Declaration of Helsinki. Informed consent was obtained from the parents or legal guardians of each participant. Between July 2020 and February 2021, eligible children with congenital and developmental cataracts, who had undergone surgery in the past three months, were prospectively enrolled. Participants were identified from pediatric ophthalmology outpatient clinics. Finally, a total of 178 participants were approached, with 166 being included in this study. Only 12 families declined participation as they did not have enough time to complete the questionnaires.

### Postoperative Children With Congenital and Developmental Cataracts

Among the participants, 69 were 0–4 years old and 38 were 5–11 years old. The following exclusion criteria were utilized: those with systemic abnormalities, secondary glaucoma, or other severe associated ocular complications, and those who had undergone ophthalmic surgery (incisional or laser) in the past one month. Additionally, parents who were unable to respond were excluded.

We gave each parent the age-appropriate questionnaire and answered their concerns. Additionally, we collected participants' demographic characteristics, including their age, gender, place of residence, and highest level of education from the parent who filled in the questionnaire. Additional clinical characteristics were obtained from their medical notes, including the eyes, staging operation, postoperative timing, glasses, amblyopia treatment, postoperative nystagmus, and postoperative strabismus.

### Visually Healthy Control

Additionally, 28 (0–4 years old) and 31 children (5–11 years old), with normal visual acuity (best-corrected visual acuity was 0.17logMAR or better at 3 years of age, 0.08logMAR or better at 4 years of age, 0.02logMAR or better at 5 years of age, and 0.0logMAR or better at 6 years or older) (7–9), no refractive corrections, and no history of eye diseases or surgeries, were enrolled as a control.

### Questionnaires

In each of the clinics, we asked one parent of each child to complete both the Proxy and Parent PedEyeQ's on paper. However, some postoperative and normal groups were located across China. Thus, these parents completed questionnaires electronically on either a mobile phone or their personal computer.

### Pediatric Eye Questionnaire

The PedEyeQ (5) includes the following sections: Child, Proxy, and Parent 0–4 PedEyeQ, as well as the Child, Proxy, and Parent 5–11 PedEyeQ. They all have distinct and separately scored domains. For responses, they each use a 3-point frequency scale (“Never,” “Sometimes,” and “All of the time”).

The Proxy 0–4 PedEyeQ contains the Functional Vision, Bothered by Eyes/Vision, and Social domains. The Proxy 5–11 PedEyeQ is composed of five domains: Functional Vision, Frustration/Worry, Social, Bothered by Eyes/Vision, and Eye Care. Additionally, the Parent PedEyeQ is composed of four domains: Worry About Their Child's Visual Function, Impact on the Parent/Family, Worry About Their Child's Self-Perception and Interactions, and Worry About Their Child's Eye Condition.

### Analysis

Statistical analyses were performed using SPSS 26.0. As mentioned, the Rasch-calibrated PedEyeQ domain scores were used to convert the data from 0 (worst) to 100 (best) for each participant. Thus, in the primary analysis, we used a Mann-Whitney *U* test to compare the differences between the children who had undergone congenital and developmental cataract surgery and the controls. Furthermore, proxy parent differences were also tested. In the secondary analyses, both Mann-Whitney U and Kruskal-Wallis tests were conducted to analyze the determinants contributing to these disparities. Values of *p* ≤ 0.05 were considered statistically significant. Furthermore, we calculated mean differences using a 95% Confidence Interval (CI) so that our estimates were more precise. PASS 15.0 (NCSs, USA) was used to calculate the sample size. The score of kids' PedEyeQ was taken as the main parameter. The calculated sample size is 13 eyes in each group. Considering the loss of follow-up rate is 20%, at least 17 eyes in each group.

## Results

### Postoperative Children With Congenital and Developmental Cataracts

Of the participants, 69 (64.5%) were aged 0 to 4 years (median: 3 years; interquartile range: 2 years) and 38 (35.5%) were aged 5 to 11 years (median: 6 years; interquartile range: 3 years). Fifty-nine children with no visual impairment (caused by refractive error, amblyopia, strabismus, and astigmatism) or eye diseases were then enrolled as the control. Twenty-eight of them (47.5%) were aged 0 to 4 years (median: 2.5 years; interquartile range: 2.5 years) and 31 (52.5%) were aged 5 to 11 years (median: 7; interquartile range: 3 years).

The demographics and clinical characteristics of the different age groups are shown in Table 1. Of the 69 0–4-year-old postoperative participants, 23 (33.33%) developed nystagmus, 39 (56.52%) developed strabismus, and 58 (84.06%) developed amblyopia. Of the 38 postoperative children aged 5–11 years, 9 (23.68%) developed nystagmus, 21 (55.26%) developed strabismus, and 28 (73.68%) developed amblyopia.

**Table 1:** Demographics and clinic characteristics of children in different ages completing the Pediatric Eye Questionnaire and their parents.

**Parameters**		**Cataract**		**Controls**	
**Groups**		**0–4 years old (*n* = 69)**	**5–11 years old (*n* = 38)**	**0–4 years old (*n* = 28)**	**5–11 years old (*n* = 31)**
**Demographic characteristics**					
Age (M, Q)	(3, 2)	(6, 3)	(2.5, 2.5)		(7, 3)
Gender of Child (NO. %)	Female	28 (40.58)	15 (39.47)	13 (46.43)	13 (41.94)
Parent/legal guardian Age	Under 25	4 (5.80)	0 (0.00)	0 (0.00)	0 (0.00)
(NO. %)	26–30	27 (39.13)	2 (5.26)	10 (35.71)	3 (9.68)
	31–35	25 (36.23)	17 (44.74)	11 (39.29)	9 (29.03)
	36–40	12 (17.39)	12 (31.58)	6 (21.43)	17 (54.8)
	over 41	1 (1.45)	7 (18.42)	1 (3.57)	2 (6.45)
Parent/legal guardian completing	Father	22 (31.88)	10 (26.32)	15 (53.57)	12 (38.7)
questionnaires (NO. %)	Mother	47 (68.12)	26 (68.42)	13 (46.43)	19 (61.2)
	Legal guardian	0 (0.00)	2 (5.26)	0 (0.00)	0 (0.00)
Place of Residence	City (NO. %)	26 (37.68)	21 (55.26)	20 (71.43)	24 (77.4)
Parent/legal guardian highest	Primary school graduate	6 (8.70)	2 (5.26)	0 (0.00)	1 (3.23)
level of education (NO. %)	Junior-high graduate/technology secondary school graduate	15 (21.74)	8 (21.05)	1 (3.57)	1 (3.23)
	high school graduate/junior college degree	24 (34.78)	12 (31.58)	2 (7.14)	4 (12.90)
	College graduate	24 (34.78)	14 (36.84)	16 (57.14)	19 (61.2)
	Postgraduate/professional degree	0 (0.00)	2 (5.26)	9 (32.14)	6 (19.35)
**Clinic characteristics**					
Eyes (NO. %)	Unilateral	20 (28.99)	14 (36.8)	NA	NA
	Bilateral	49 (71.01)	24 (63.2)	NA	NA
Staging operation (NO. %)	Primary IOL implantation	23 (33.33)	26 (68.42)	NA	NA
	Aphakia	21 (30.43)	4 (10.53)	NA	NA
	Secondary IOL implantation	25 (36.23)	8 (21.05)	NA	NA
Postoperative timing (NO. %)	<1 year	16 (23.19)	3 (7.89)	NA	NA
	1 to 2 years	18 (26.09)	3 (7.89)	NA	NA
	2 to 3 years	13 (18.84)	6 (15.79)	NA	NA
	More than > 3 years	22 (31.88)	26 (68.42)	NA	NA
Glasses (NO. %)	Wear glasses	68 (98.55)	38 (100.00)	NA	NA
Amblyopia treatment (NO. %)	Cooperative	18 (26.09)	17 (44.74)	NA	NA
	Average	28 (40.58)	11 (28.95)	NA	NA
	Uncooperative	12 (17.39)	0 (0.00)	NA	NA
	No treatment	11 (15.94)	10 (26.32)	NA	NA
Postoperative nystagmus (NO. %)	With nystagmus	23 (33.33)	9 (23.68)	NA	NA
Postoperative strabismus (NO. %)	With strabismus	39 (56.52)	21 (55.26)	NA	NA

### Comparing PedEyeQ Scores in Postoperative Children and the Control Group

#### Proxy PedEyeQ

**Figure 1:**
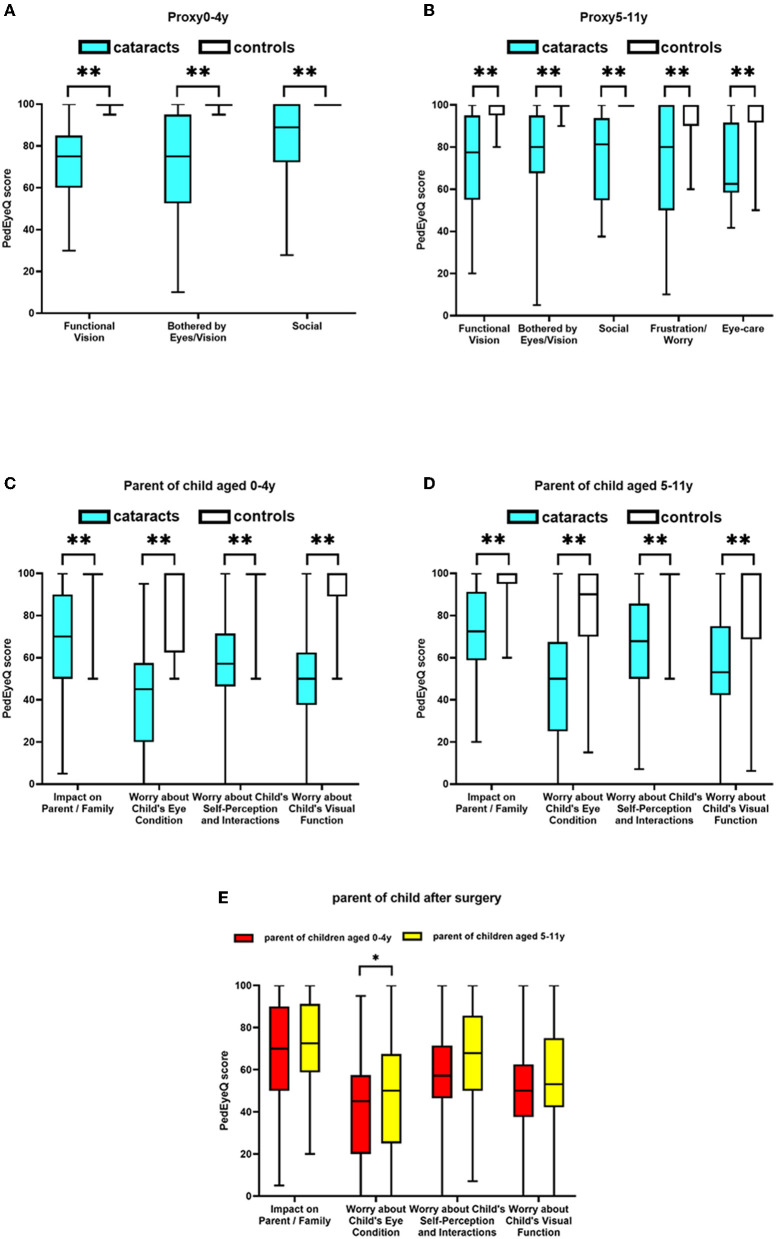
PedEyeQ domain scores in children following congenital and developmental cataracts surgery compared to healthy controls; **(A)** Proxy PedEyeQ scores for children aged 0–4 years; **(B)** Proxy PedEyeQ scores for children aged 5–11 years; **(C)** PedEyeQ scores for parents of children aged 0–4 years; **(D)** PedEyeQ scores for parents of children aged 5–11 years. **(E)** Comparison of PedEyeQ scores for parents of children after surgery. * means the results between two groups have significant difference statistically and ** means a greater significant difference.

The postoperative children aged 0- to 4-years-old had lower scores than the controls on each of the three Proxy 0–4 years PedEyeQ domains (*p* < 0.001; Table 2; Figure 1A). The PedEyeQ Proxy scores for participants aged 0 to 4 years' greatest mean difference was 27 points worse in the Functional Vision domain (median 75 vs. 100; mean difference −27; 95% CI −34 to −19; *p* < 0.001; Table 2; Figure 1A).

**Table 2:** PedEyeQ domain scores for kids' age among 0–4 with congenital cataracts (*N* = 69), age among 0–4 normal controls (*N* = 28), age among 5–11 with congenital cataracts (*N* = 38), age among 5–11 normal controls (*N* = 31).

**Proxy PedEyeQ domains**	**Median (range) Proxy PedEyeQ scores**		***P* value for difference Cataracts vs. Controls**	**Mean difference (95% CI) Cataracts vs. Controls**
	**Congenital cataracts**	**Controls**		
**Age 0–4 PedEyeQ domains**	***N*** **=** **69**	***N*** **=** **28**	
Functional Vision	75 (30–100)	100 (95–100)	**<0.001**	−27 (−34 to −19)
Bothered by eyes/vision	75 (10–100)	100 (95–100)	**<0.001**	−19 (−35 to −27)
Social	89 (28–100)	100 (100–100)	**<0.001**	−17 (−35 to −19)
**Age 5–11 PedEyeQ domains**	***N*** **=** **38**	***N*** **=** **31**		
Functional Vision	78 (20–100)	100 (80–100)	**<0.001**	−23 (−30 to −15)
Bothered by eyes/vision	80 (5–100)	100 (90–100)	**<0.001**	−21 (−29 to −14)
Social	81 (37–100)	100 (100–100)	**<0.001**	−23 (−31 to −16)
Frustration/worry	80 (10–100)	100 (60–100)	**<0.001**	−20 (−30 to −9)
Eye-care	63 (42–100)	100 (50–100)	**<0.001**	−21 (−29 to −13)
**Parents of Age 0–4 PedEyeQ domains**	***N*** **=** **69**	***N*** **=** **28**		
Impact on parent/family	70 (5–100)	100 (50–100)	**<0.001**	−28 (−38 to −18)
Worry about child's eye condition	45 (0–95)	100 (50–100)	**<0.001**	−46 (−57 to −36)
Worry about child's self-perception/interactions	57 (0–100)	100 (50–100)	**<0.001**	−34 (−45 to −23)
Worry about child's visual function	50 (0–100)	100 (50–100)	**<0.001**	−40 (−45 to −23)
**Parents of Age 5–11 PedEyeQ domains**	***N*** **=** **38**	***N*** **=** **31**		
Impact on parent/family	72 (20–100)	100 (60–100)	**<0.001**	−22 (−30 to −14)
Worry about child's eye condition	50 (0–100)	90 (15–100)	**<0.001**	−29 (−43 to −16)
Worry about child's self-perception/interactions	68 (7–100)	100 (50–100)	**<0.001**	−26 (−37 to −16)
Worry about child's visual function	53 (0–100)	100 (6–100)	**<0.001**	−30 (−43 to −17)

*Bold values indicate significant difference between two groups*.

In the Proxy 5–11 years PedEyeQ, the postoperative children aged 5–11 years had worse scores across each of the five domains compared to the controls (*p* < 0.001; Table 2; Figure 1B). The greatest difference was in the Functional Vision domain (median 78 vs. 100; mean difference −23; 95% CI −30 to −15; *p* < 0.001; Table 2; Figure 1B). Additionally, nystagmus was the main determinant herein (*p* < 0.05).

#### Associations

Demographics and clinical characteristics were analyzed using the Mann-Whitney *U* test. Nystagmus (*p* < 0.005; Table 3) and strabismus (*p* < 0.005; Table 3) were found to be the most significant clinical factors affecting participants' functional vision. In particular, male children (*p* < 0.001; Table 3) and female parents (*p* < 0.05; Table 3) had lower scores in the Functional Vision domain. Additionally, the more uncooperative that children were to undergo amblyopia treatment, the lower their scores (*p* < 0.05; Table 3).

**Table 3:** Dominating factors on functional vision of age 0–4 and 5–11 PedEyeQ domains.

**Parameters**	***P* value of children's gender**	***P* value of parents' gender**	***P* value of Amblyopia treatment**	***P* value of Eyes**	***P* value of Nystagmus**	***P* value of Strabismus**
**Age 0–4 PedEyeQ domains**						
Functional Vision	**0.001**	**0.039**	**0.035**	0.422	**0.002**	**0.003**
Bothered by eyes/vision	**0.002**	0.085	**0.030**	0.979	**0.011**	**0.008**
Social	0.119	**0.047**	**0.013**	0.315	**0.006**	0.234
**Age 5–11 PedEyeQ domains**						
Functional Vision	0.708	0.534	0.099	0.386	**0.035**	0.376
Bothered by eyes/vision	0.916	0.580	0.432	0.680	**0.033**	0.625
Social	0.893	0.364	0.552	0.595	0.469	0.690
Frustration/worry	0.938	0.343	0.994	0.254	0.136	0.940
Eye-care	0.211	0.734	**0.023**	0.593	**0.002**	**0.033**

*Bold values indicate significant difference among groups*.

#### Parent PedEyeQ

Parents of children aged 0–4 years' PedEyeQ scores were lower in each domain for those of postoperative children compared to parents of those in the control (*p* < 0.001; Table 2; Figure 1C). Parent PedEyeQ 0 to 4 years' greatest difference was 46 points worse on Worry About Their Child's Eye Condition domain (median 45 vs. 100; mean difference −46; 95% CI −57 to −36; *p* < 0.001; Table 2; Figure 1C). Parents of patients with nystagmus (*p* < 0.001) or strabismus (*p* < 0.05) were significantly more worried about their children's eye condition.

In the Parent PedEyeQ, parents of children aged 5–11 years also reported worse scores across each of the four domains compared to those of the controls (*p* < 0.001; Table 2; Figure 1D). Parents responding to the PedEyeQ 5 to 11 years' greatest difference was 30 points worse on the Worry About Their Child's Visual Function domain (median 53 vs. 100; mean difference −30; 95% CI −43 to −17; *p* < 0.001; Table 2; Figure 1D). Nevertheless, the demographic and clinical factors had only small effects on participants' functional vision.

#### Comparing PedEyeQ Scores in Parents of Children Aged 0–4 vs. Those of Children Aged 5–11 Years

Parental PedEyeQ scores of those with children aged 0 to 4 years showed the greatest difference in their 10 points lower scores on the Worry About Their Child's Eye Condition Domain (median 45 vs. 50; mean difference −10; 95% CI −21 to 0; *p* < 0.05; Table 4; Figure 1E) compared to those of children aged 5–11 years.

**Table 4:** PedEyeQ domain scores for kids' age among 0–4 with congenital cataracts (*N* = 69), age among 5–11 with congenital cataracts (*N* = 38).

**Proxy PedEyeQ domains**	**Median (range) Proxy PedEyeQ scores**		***P* value for difference Group 0–4 y vs. Group 5–11 y**	**Mean difference (95% CI) Cataracts vs. Controls**
	**0–4 y children of congenital cataracts**	**5–11 y children of congenital cataracts**		
**Parents PedEyeQ domains**	***N*** **=** **69**	***N*** **=** **38**		
Impact on parent/family	70 (5–100)	72 (20–100)	0.24	−6 (−15 to 4)
Worry about child's eye condition	45 (0–95)	50 (0–100)	**0.045**	−10 (−21 to 0)
Worry about child's self-perception/interactions	57 (0–100)	68 (7–100)	0.091	−9 (−20 to 1)
Worry about child's visual function	50 (0–100)	53 (0–100)	0.327	−5 (−17 to 6)

*Bold values indicate significant difference between two groups. y=years old*.

## Discussion

This study was the first to use the PedEyeQ to examine the quality of life (QOL) of both postoperative children with congenital and developmental cataracts and their parents. Furthermore, we found that appearance abnormalities, such as nystagmus and strabismus, significantly influenced their QOL.

The impact of reduced functional visual abilities of children with congenital and developmental cataracts has seldom been evaluated in previous studies. Tailor et al. (10) used the Cardiovascular Visual Ability Questionnaire for Children (CVAQC) to measure the functional visual abilities of children treated for cataracts. They recorded the putative risk factors herein, such as visual acuity and the number of previous surgical interventions. However, they did not explore the associations between participants' functional vision impairments and these factors. Furthermore, they measured these factors using three validated instruments, which complicates their findings. Paryani et al. (11) found low scores in children who had undergone an operation for cataracts, especially bilateral cataracts. They used the LV Prasad Functional Vision Questionnaire (LVP-FVQ). However, both the CVAQC and the LVP-FVQ were specifically designed for children with vision impairment and not for all eye conditions (12–14).

Chak et al. (4) used the PedsQL 4.0 to assess the health-related QOL (HRQOL) in children with congenital cataracts. Their HRQOL scores were found to be significantly impacted, which were comparable with those of children with systemic chronic, severe, or life-threatening diseases. However, the current literature still lacks sufficient content on eye condition impairments.

There are several questionnaires that measure ER-QOL; however, they primarily target adolescents and young adults (13, 15–17). Due to improvements in medical technologies, the youngest viable age for undergoing pediatric cataract surgery has increased. Furthermore, undergoing surgery at a younger age is more likely to result in better visual outcomes (18, 19). Some experts have even suggested that surgery should be performed within 6–8 weeks of birth (20). Thus, more research is needed on the impact of these surgeries on younger children.

PedEyeQ, with its distinct domains, provides a means with which to compensate for these research gaps. The PedEyeQ is an accurate instrument that has been developed to assess the impact of children's eye conditions on both them and their caregivers. The Child, Proxy, and Parent pediatric eye questionnaires of the PedEyeQ were designed separately and can be used across a wide age range of respondents. Additionally, the PedEyeQ's Rasch scoring system is convenient as its frequency scale and question format are easily understood (21). Finally, it has more items than most other measures and can simultaneously examine respondents' functional vision and ER-QOL.

The factor structure and validity, as well as the known-group, discriminant, and convergent validities, of the PedEyeQ have been established in studies of children with bilateral visual impairment (6), residual amblyopia (22), strabismus (23), unilateral visual acuity deficit (24), and correlational research between functional measures and the Child PedEyeQ (25).

The present study confirms previous publications (26). Using the PedEyeQ, Leske et al. (26) reported reduced functional vision and ER-QOL across various age groups and a wide range of pediatric eye conditions. A total of 1,037 patients with pediatric eye conditions (cataract, *n* = 99) and 254 visually healthy controls were included in their study. However, specific eye conditions, such as congenital cataracts, still need further study. Furthermore, only 16% of the 1,037 patients were in active treatment, meaning that changes in their response to treatments, like surgery, may impact both their functional vision and ER-QOL. Thus, the children we recruited were those who had undergone congenital and developmental cataract surgery. Additionally, we further explored the associations of the study factors with demographic and clinical factors.

The Proxy PedEyeQ domain scores were significantly worse for the participants compared to those of the controls. This shows that postoperative children have reduced functional vision and ER-QOL. Using the Parent PedEyeQ, we also discovered that, unlike parents of visually healthy children, those of postoperative children experience a lower QOL. Our findings are consistent with those of prior research (3, 4, 11, 26–28).

In our research, the greatest mean differences were in the Functional Vision domain. This is indubitable given the impaired visual acuity and poor eye condition found in the cataract group. The Functional Vision domain of the Proxy PedEyeQ focuses on children's daily activities and school-related tasks. Postoperative children with congenital and developmental cataracts are more likely to showcase slower reading skills and a reduced physical.

We further analyzed the determinants contributing to this outcome using Mann-Whitney U and Kruskal-Wallis tests. Demographic and clinical characteristics were both considered herein. Finally, we found that having either nystagmus or strabismus was the major clinical factor affecting participants' Functional Vision domain. Additionally, more willing cooperation during amblyopia treatment (0–4 years, *p* < 0.05; 5–11 years, *p* = 0.099) and the staging operation (5–11 years, *p* = 0.090) are important factors influencing respondents' functional vision. However, likely because of our small sample size, these two variables were not statistically significant.

Our findings accurately outline the clinical reality of postoperative children with congenital and developmental cataracts. For example, we found that nystagmus, strabismus, and amblyopia are all likely to have an impact on peoples' daily activities (22, 28, 29) because reduced visual acuity and stereoacuity all influence their functional vision. Patients with amblyopia are more likely to have a slower reading speed (30), with those with strabismus being more likely to also exhibit motor skill deficits (30). Furthermore, having physically noticeable strabismus (31) or nystagmus (32) is often problematic for school children as others may treat them as “different.” Thus, children's well-being and developmental progress are affected by these conditions. Likewise, needing to wear specific glasses or an eye patch due to these conditions can also draw negative attention to children with amblyopia form their peers (33).

Our findings are in accordance with prior studies on children with various eye conditions. For example, a series of studies by the American Pediatric Eye Disease Investigator Group reported that the PedEyeQ greatest mean differences are worse in the Functional Vision domain for patients with these conditions. However, they directly explored the association of specific eye conditions, such as strabismus (23), amblyopia (22), and nystagmus (26), with functional vision and ER-QOL in their participants. This means that nystagmus, strabismus, and amblyopia are significant factors in this relationship.

Notably, we compared functional vision and ER-QOL changes in postoperative children with unilateral and bilateral cataracts; however, no statistically significant difference was found. In contrast, other studies have reported that bilateral pediatric cataracts provide better visual functioning than unilateral ones (11, 34–37). However, this discrepancy is likely because both the unilateral and bilateral cataracts used in our study were of congenital or developmental etiology, while the unilateral cataracts used in the prior research were traumatic ones. Due to an uneven visual deprivation, the prevalence of amblyopia (100% vs. 77.6%) and strabismus (65.0% vs. 53.1%) was slightly higher after participants had undergone unilateral cataract surgery in our study. However, the incidence of complications following traumatic cataract surgery was higher (34). Additionally, this type of unilateral cataracts' vision and VQL are much lower.

In addition to the demographic and clinical characteristics included in our study, there are still other factors impacting patients' functional vision, meaning that more research is needed.

Parents of children aged 0–11 years had significantly negative PedEyeQ domain scores. The greatest mean differences were in the Worry About Their Child's Eye Condition and the Worry About Their Child's Visual Function domains. Specifically, the scores were significantly lower for parents of 0–4 year old children following congenital and developmental cataracts surgery in the Worry About Their Child's Eye Conditions domain (*p* < 0.05). Furthermore, in previous research (6, 22, 23) that also used the PedEyeQ to study various eye conditions, the greatest mean differences were observed in these same two domains. After an analysis of certain factors, we found that parents of children with nystagmus (0–4 years, *p* < 0.001) or strabismus (0–4 years, *p* < 0.05) were significantly more worried about their children's eye condition. Our findings indicate that parents tend to overestimate the influence of nystagmus and strabismus on their children following surgery. Additionally, it has been documented that patients with nystagmus or strabismus have worsened stereo functioning and control (38). Furthermore, it is likely that pediatric patients do not openly communicate with their parents about their school life, which then adds to the latter's psychological concerns (39). For example, they may worry about their children's learning and social abilities (40). Moreover, if the children are very young, their treatments will need to start as early on as possible, which may then add to their parents' financial pressure. Finally, long-term expectations and their understanding of their children's health may also affect parents' QOL.

We also found that parent PedEyeQ scores of those with children aged 0 to 4 years were generally lower than those of children aged 5 to 11 years. It is likely that their children's poor eye condition is the cause because, when compared with postoperative children aged 5–11 years, the 0–4 year old ones had a higher prevalence of nystagmus (33.3% vs. 23.7%), strabismus (56.5% vs. 55.3%), and amblyopia (84.1% vs. 73.7%). Our findings are similar to those reported by Birch et al. (41). They also found a high prevalence of nystagmus and strabismus in children aged 0–4 years after cataract surgery. Therefore, Parent PedEyeQ's greatest difference between these two age groups is most likely to be in the Worry About Their Child's Eye Condition domain. Perhaps our sample size was not large enough, which is why these two groups did not show any significant differences in the other three domains.

Thus, interventions to improve the QOL of postoperative children with congenital and developmental cataracts, and that of their parents, are necessary. Both patients and their parents should actively participate in therapy, especially in terms of the management of nystagmus, strabismus, and amblyopia. Applying the concept of win-win cooperation herein is also worth applying for both parties. Alternatively, engaging in outpatient follow-up is important because these children's eye conditions change with time. As such, an experienced clinician is needed to formulate the correct diagnosis and treatment plan. To ameliorate the adverse effects of the ER-QOL on the patient's family, educational interventions, such as clinical lectures, may be helpful. Psychosocial interventions and peer support groups should also be evaluated.

Because our sample size was small, future studies should use larger populations to evaluate more impact factors in this relationship. Additionally, because the child PedEyeQ we used was proxy-reported, parental perceptions may also have influenced their children's answers. Finally, this was a retrospective cross-sectional study. A prospective study is thus needed to identify any differences at various stages after surgery.

## Conclusions

Although treatments for congenital and developmental cataracts are highly successful, their effects on the QOL and functional vision of postoperative children and their families are still significant. Families and their children would benefit from receiving clinical support, educational interventions, psychosocial interventions, and peer support.

## Data Availability Statement

The original contributions presented in the study are included in the article/supplementary material, further inquiries can be directed to the corresponding author.

## Ethics Statement

The studies involving human participants were reviewed and approved by Ethics Committee of the Affiliated Ophthalmology Hospital of Wenzhou Medical University. Written informed consent from the participants' legal guardian/next of kin was not required to participate in this study in accordance with the national legislation and the institutional requirements.

## Author Contributions

The study was designed by YuZ and YiZ. Conducting of the study, data collection, and its analysis and interpretation were completed by SG, YH, LC, WS, PC, and DW. All authors contributed to the article and approved the submitted version.

## Funding

This study was supported by research grants from Natural Science Foundation of Zhejiang Province (Grant No. LQ19H120001) and National Natural Science Foundation of China (Grant No. 81870680).

## Conflict of Interest

The authors declare that the research was conducted in the absence of any commercial or financial relationships that could be construed as a potential conflict of interest.

## Publisher's Note

All claims expressed in this article are solely those of the authors and do not necessarily represent those of their affiliated organizations, or those of the publisher, the editors and the reviewers. Any product that may be evaluated in this article, or claim that may be made by its manufacturer, is not guaranteed or endorsed by the publisher.
